# Effect of atorvastatin versus no *S*tatin *T*reatment on major clinical events in *A*cute *C*ardio*E*mbolic stroke patients without a definite indication for statin therapy: protocol for the STACE trial

**DOI:** 10.1186/s13063-025-09097-x

**Published:** 2025-09-26

**Authors:** Hong-Kyun Park, Jun Yup Kim, Keun-Sik Hong, Yong-Jin Cho, Jong-Moo Park, Dongwhane Lee, Kyusik Kang, Soo Joo Lee, Jae Guk Kim, Jae-Kwan Cha, Dae-Hyun Kim, Moon-Ku Han, Beom Joon Kim, Jihoon Kang, Tai Hwan Park, Sang-Soon Park, Jin Kyo Choi, Kyungbok Lee, Jeong-Yoon Lee, Jun Lee, Doo Hyuk Kwon, Byung-Chul Lee, Kyung-Ho Yu, Mi Sun Oh, Minwoo Lee, Man-Seok Park, Joon-Tae Kim, Kang-Ho Choi, Hyunsoo Kim, Dong-Eog Kim, Dong-Seok Gwak, Jay Chol Choi, Joong-Goo Kim, Chul-Hoo Kang, Jee-Hyun Kwon, Wook-Joo Kim, Dong-Ick Shin, Kyu Sun Yum, Sung Il Sohn, Jeong-Ho Hong, Hyungjong Park, Chulho Kim, Sang-Hwa Lee, Kwang-Yeol Park, Hae-Bong Jeong, Chan-Young Park, Kyungmi Oh, Chi Kyung Kim, Jung Hoon Han, Keon-Joo Lee, Sung Hyuk Heo, Ho Geol Woo, Juneyoung Lee, Hee-Joon Bae

**Affiliations:** 1 Department of Neurology, Inje University Ilsan Paik Hospital, Inje University College of Medicine, Goyang, Republic of Korea; 2 Department of Neurology, Cerebrovascular Center, Seoul National University College of Medicine, Seoul National University Bundang Hospital, Seongnam, Republic of Korea; 3 Department of Neurology, Uijeongbu Eulji Medical Center, Eulji University School of Medicine, Uijeongbu, Republic of Korea; 4 Department of Neurology, Nowon Eulji Medical Center, Eulji University School of Medicine, Seoul, Republic of Korea; 5 Department of Neurology, Daejeon Eulji Medical Center, Eulji University School of Medicine, Daejeon, Republic of Korea; 6 Deparment of Neurology, Dong-A University Hospital, Busan, Republic of Korea; 7 Department of Neurology, Seoul Medical Center, Seoul, Republic of Korea; 8 Department of Neurology, Soonchunhyang University Hospital, Seoul, Republic of Korea; 9 Department of Neurology, Yeungnam University Hospital, Daegu, Republic of Korea; 10 Department of Neurology, Hallym University Sacred Heart Hospital, Anyang, Republic of Korea; 11 Department of Neurology, Chonnam National University Hospital, Gwangju, Republic of Korea; 12 Department of Neurology, Dongguk University Ilsan Hospital, Goyang, Republic of Korea; 13 Department of Neurology, Jeju National University Hospital, Jeju National University, College of Medicine, Jeju, Republic of Korea; 14 Department of Neurology, Ulsan University College of Medicine, Ulsan, Republic of Korea; 15 Department of Neurology, Chungbuk National University Hospital, Chungbuk National University College of Medicine, Cheongju, Republic of Korea; 16 Department of Neurology, Keimyung University Dongsan Medical Center, Daegu, Republic of Korea; 17 Department of Neurology, Hallym University Chuncheon Sacred Heart Hospital, Chuncheon, Gangwon-Do Republic of Korea; 18 Department of Neurology, Chung-Ang University Hospital, Seoul, Republic of Korea; 19 Department of Neurology, Korea University Guro Hospital, Seoul, Republic of Korea; 20 Department of Neurology, Kyung Hee University Hospital, Seoul, Republic of Korea; 21 Department of Biostatistics, Korea University College of Medicine, Seoul, Republic of Korea

**Keywords:** Acute ischemic stroke, Cardioembolism, Statin, Guidelines

## Abstract

**Background:**

Evidence supporting the use of statin therapy to reduce stroke recurrence and cardiovascular events in acute cardioembolic stroke (CES) patients without atherosclerosis is limited. Past observational studies have been hampered by selection bias and unmeasured confounding factors. This study aims to investigate the potential benefits of statin therapy in acute CES patients without established indications through a registry-based, randomized clinical trial.

**Methods:**

This is a registry-based, multicenter, prospective, randomized, open-label, blinded endpoint (PROBE) study designed to evaluate the efficacy and safety of statin therapy in acute CES patients without established indications for statin use. Patients will be randomly assigned (1:1) to either statin users or non-users, with statin users receiving atorvastatin at a dose of 10 mg or higher throughout the study period. We plan to recruit 1036 participants to detect a relative risk reduction of 43% with 80% power and a two-sided alpha error of 0.05, accounting for a 10% loss to follow-up. The primary outcome is the occurrence of a major clinical event, defined as a composite of stroke recurrence, myocardial infarction, and all-cause mortality within 3 months after the index stroke. The secondary efficacy outcomes include (1) stroke recurrence, (2) all-cause mortality, (3) vascular death, and (4) major vascular events.

**Discussion:**

This study will assist stroke physicians in determining the appropriate use of statin therapy for acute CES patients who do not have guideline-based indications.

**Trial registration:**

CRIS Registration Number: KCT0006806. Registered on November 29, 2021. URL: https://cris.nih.go.kr/cris

**Supplementary Information:**

The online version contains supplementary material available at 10.1186/s13063-025-09097-x.

## Background

Cardioembolic stroke (CES) is one of the most severe and fatal subtypes of ischemic stroke, often resulting in high early mortality and poor long-term outcomes [1, 2]. However, there remains a lack of evidence regarding optimal secondary prevention strategies, particularly for patients who do not meet current statin eligibility criteria. Despite advances in stroke prevention and treatment, optimal strategies for secondary prevention in CES remain unclear—particularly for patients who fall outside current guideline-based statin eligibility. While high-intensity statins have shown benefit in reducing recurrent events among patients with recent ischemic stroke or transient ischemic attack (TIA) without known coronary heart disease (CHD) [3], the role of statin therapy in CES patients without a clear indication remains uncertain and under-investigated.

Current guidelines remain uncertain regarding the benefits of statin use in patients without established atherosclerotic disease [4]. The inconsistency between guidelines and related performance measures for statin use in ischemic stroke patients further complicates the issues, potentially leading to the inappropriate use of statins in this population [4–7]. For patients with cardioembolic stroke (CES), evidence regarding statin use is particularly lacking, especially in those without concomitant atherosclerosis. The Stroke Prevention by Aggressive Reduction in Cholesterol Levels (SPARCL) trial excluded patients with cardiac sources of embolism (e.g. CHD, atrial fibrillation, prosthetic heart valve, significant mitral stenosis, and sinus node dysfunction) [3]. Observational studies have suggested that statin therapy may reduce mortality and vascular events in CES patients [8, 9]. However, these studies have important limitations. One included patients with conditions known to benefit from statins, such as aortic atheroma or coronary artery disease [9], potentially introducing selection bias. Another did not clearly report whether patients with established indications for statin therapy were excluded [8], making it difficult to isolate the effect of statin in CES patients with such indications.

In a recent study using the nationwide, multicenter, prospective stroke registry in Korea (Clinical Research Collaboration in Korea-National Institute of Health; CRCS-K-NIH registry), statin therapy was associated with decreased mortality and vascular events in patients with acute CES, even though these patients had no established indication for statin therapy according to current guidelines [10]. Despite these findings, the retrospective design of the study, the presence of residual or unmeasured confounding factors, and inherent biases limit the generalizability of its conclusions.

This study aims to evaluate the potential benefits of statin therapy in reducing major clinical events within 90 days among patients with acute cardioembolic stroke who do not meet current guideline-based indications for statin use.

## Methods

### Study design

The Statin Therapy for the Prevention of Major Clinical Events in Acute CardioEmbolic Stroke Patients without a Definite Indication for Statins (STACE) Trial is a registry-based, multicenter, prospective, randomized, open-label, blinded end-point (PROBE) study. This study aims to evaluate the efficacy and safety of statin therapy in acute CES patients who have no established indication for statin therapy according to current guidelines within a registry-based randomized clinical trial (RRCT) setting.

The RRCT design offers several advantages in clinical research. By utilizing real-world patient data, it ensures diverse patient representation, thereby enhancing the generalizability of the findings [11]. Integrating randomization within registry frameworks increases trial efficiency, minimizes recruitment challenges, and reduces administrative costs. Additionally, since registry data are collected as part of routine clinical practice, the effort required to capture outcomes is reduced compared to traditional RCTs. Overall, the registry-based RCT approach provides valuable insights into treatment effectiveness and safety in real-world settings, offering a cost-effective and efficient method for conducting clinical research.

**Fig. 1:**
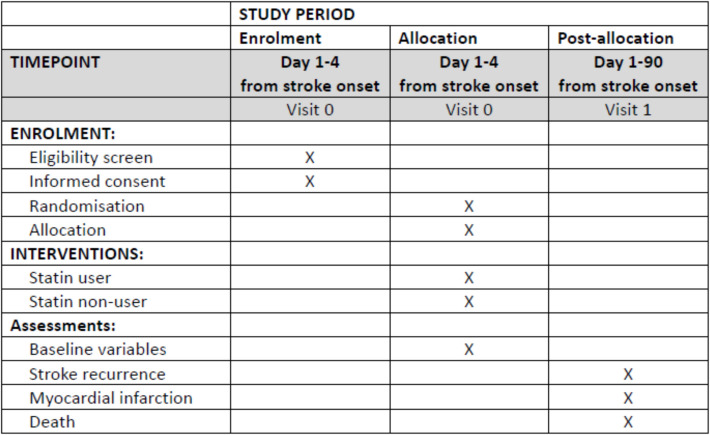
STACE study timeline

This RRCT utilizes the CRCS-K-NIH registry for patient enrollment, randomization, outcome assessment, and follow-up. Study timeline conforming to the SPIRIT guidelines is in Fig. 1. Detailed information about the CRCS-K-NIH registry was published previously [12] and is described in the Supplemental Methods.

### Patient population—inclusion and exclusion criteria

Adults (age of 19 or older) with acute ischemic stroke (within 4 days of symptom onset) of the cardioembolic subtype, as classified by a modified Trial of Org 10,172 in Acute Stroke Treatment classification system [13], are eligible for the study. High-risk cardioembolic sources [13] are listed in the Supplemental Table 1. Patients who were taking statins on a regular basis prior to the index stroke will not be included in the study.

**Fig. 2:**
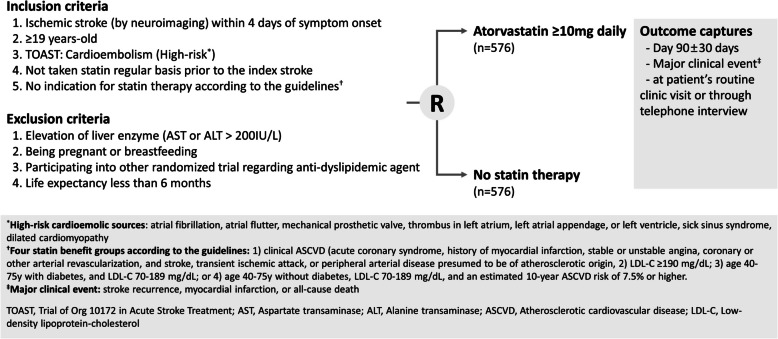
Study flowchart

Patients who do not meet any of the criteria for the four statin benefit groups, as defined by the 2013 American College of Cardiology (ACC)/American Heart Association (AHA) Cholesterol guidelines, are eligible for this trial (Fig. 2) [4]. The four statin benefit groups are summarized as follows: (1) individuals with clinical atherosclerotic cardiovascular disease (ASCVD); (2) individuals with elevated low-density lipoprotein-cholesterol (LDL-C) levels ≥ 190 mg/dL; (3) individuals aged 40–75 years with diabetes and LDL-C levels between 70 and 189 mg/dL; or (4) individuals without diabetes, aged 40–75 years, with LDL-C levels between 70 and 189 mg/dL and an estimated 10-year ASCVD risk of 7.5% or higher. The determination of statin indications will be based on information obtained at the time of hospitalization for the index stroke.

Patients with any of the following criteria will be excluded from the study: (1) refusal to participate in the study, (2) abnormal liver function test (aspartate transaminase or alanine transaminase > 200 IU/L), (3) contraindications to or allergies to statins, (4) pregnancy or breast-feeding, (5) participation in another clinical trial involving anti-dyslipidemic agents, (6) a life expectancy of 6 months or less, or (7) use of contraindicated medications, such as other statins, proprotein convertase subtilisin/kexin type 9 inhibitors, cyclosporine, or fibrates.

After verification of the inclusion and exclusion criteria, written informed consent will be obtained from the patients. Patients will be informed about the purpose, process, and the expected benefit and harm of participation.

### Randomization

Once a patient provides informed consent to participate in the study, they will be randomly assigned to either the statin user group or the statin non-user group through the Interactive Web Response System (IWRS) integrated within the CRCS-K-NIH/STACE registry database. Randomization will be conducted using permuted block randomization with a pre-specified block size, stratified by participating centers to ensure balanced allocation across sites. The randomization sequence will be pre-generated by an independent biostatistician, and randomization will occur within four calendar days of symptom onset.

Although the trial is open-label, allocation concealment was strictly maintained. The pre-generated randomization sequence was embedded in a centralized IWRS implemented on the CRCS-K-NIH registry platform. This system ensures that treatment assignments remained concealed from both investigators and participants until the point of allocation, thereby minimizing selection bias and preserving the integrity of the randomization process.

In addition, patients who meet the eligibility criteria but decline participation will be monitored and recorded within the registry database; these individuals will comprise the non-RRCT analysis population, as defined in the Statistical Analysis section.

### Intervention

Following randomization to the treatment group (statin user) or the control group (statin non-user), clinical trial medication will be administered within four calendar days of symptom onset. Patients assigned to the statin user group will receive atorvastatin, either orally or via a nasogastric tube. A daily dose of atorvastatin 10 mg or higher was chosen to reflect clinical practice in Korea, where moderate- to high-intensity statin is commonly prescribed. A recent study from Korea [10] showed that most patients were prescribed moderate-to-high intensity statins (high intensity, 35%; moderate intensity, 65%; and low intensity, 5%), following the ACC/AHA 2013 Blood Cholesterol Management Guidelines [4] in acute CES patients. Subjects in the treatment group will take the clinical trial medication for 3 months, after which the continuation of statin therapy will be determined by their physicians in charge. In the control group, initiation of statin therapy will be at the discretion of the attending physicians after the initial 3-month period.

### Outcomes

The primary outcome is an occurrence of a major clinical event, defined as a composite of stroke recurrence, myocardial infarction, and all-cause death within 3 months of the index stroke. Secondary efficacy outcomes are (1) stroke recurrence, (2) all-cause death, (3) vascular death, and (4) major vascular events (comprising stroke recurrence, myocardial infarction, and vascular death). Secondary safety outcomes are (1) hemorrhagic stroke and (2) symptomatic hemorrhagic transformation.

In accordance with the predefined outcome capture protocol of the CRCS-K-NIH registry, a clinical research coordinator will prospectively collect outcome events during hospitalization and at the 3-month follow-up, either at patients’ routine clinic visits or through telephone interviews with the patients themselves or their caregivers. To ensure consistency in outcome capture during interviews, a set of uniform structured questionnaires will be utilized (Supplemental Table 2). Detailed definitions of each outcome are provided in Supplemental Table 3.

### Data and Safety Monitoring Board

The Data and Safety Monitoring Board (DSMB) of this trial is composed of four members (three stroke physicians and one independent biostatistician) who are not involved in the trial’s implementation. The primary objective of the DSMB is to periodically review safety data and determine whether the trial has reached a point where discontinuation may be warranted due to demonstrated efficacy or futility.

### Sample size estimates

To estimate event rates, a simulation study was performed using the CRCS-K-NIH registry database to closely reflect the characteristics of the trial’s target population. Data from a total of 5,261 patients who met the study’s selection criteria between April 2008 and November 2019 were extracted from the registry. During this period, the registry included 72,796 patients, of whom 65,801 had ischemic stroke, 63,340 presented within 7 days of symptom onset, 13,853 had cardioembolic stroke, 5579 did not receive statin therapy, and 5261 arrived at the hospital within 2 days of symptom onset.

The timing of randomization was based on a recent Korean study which showed that a significant number of major vascular events associated with statin therapy occur within the first month, particularly within the first 7 days [10]. To account for clinical feasibility, five randomization scenarios were developed (Supplemental Table 4), and scenario #3 was selected for simulation. Major event rates within 90 days between the treatment and control groups were compared for each of the 10 simulated datasets (Supplemental Table 5).

Considering that stroke severity is closely related to clinical outcomes, patients were categorized into three groups based on the National Institutes of Health Stroke Scale scores: mild (0–4), moderate (5–10), and severe (> 10). The estimated between-group differences in 90-day event rates were 2% for the mild group, 5% for the moderate group, and 25% for the severe group. These findings suggest that the overall 17% risk difference may have been driven disproportionately by the severe group, where indication bias and unmeasured confounders could have affected statin use and outcomes.

To minimize this potential bias, we based our sample size calculation on the moderate severity group. Using event rates of 7% in statin users and 12% in non-users (hazard ratio of 0.568), we estimated that 1036 patients and 99 events would provide 80% power to detect a 43.2% relative risk reduction at a two-sided alpha of 0.05, based on a group sequential log-rank test. An interim analysis at 50% of the information time is conducted using the O’Brian-Flemming type alpha spending function. Accounting for a 10% loss to follow-up, we plan to enroll 1152 patients under the intention-to-treat (ITT) principle.

### Statistical analysis

A log-rank test will be performed to test the primary hypothesis that statin users will have a lower incidence of major clinical events within 3 months compared to non-users. The same analysis will also be applied to assess secondary outcomes. Cox proportional hazard models will be used to adjust for any imbalances in baseline variables between the groups. The intention-to-treat (ITT) population is defined as all subjects who meet eligibility criteria and are hence randomized, while the per-protocol (PP) population consists of those who have more than 80% medication adherence and are assessed for the primary efficacy outcome among the ITT population. Subjects who take at least one medication after randomization will be classified as the safety population. While safety outcomes will be assessed using the safety population, both ITT and PP analyses will be performed for efficacy outcomes, but the results of the ITT analysis will be served as the primary determinant of trial outcomes.

We further define a non-RRCT population as individuals from the CRCS-K-NIH registry who meet trial eligibility criteria but choose not to participate. A comparison of baseline characteristics and outcomes between the RRCT ITT population and the non-RRCT population will be made to identify any significant imbalances in clinical characteristics between trial participants and nonparticipants.

An interim analysis will be performed at the time of 50 events, representing 50% of the expected primary efficacy endpoint events that have occurred. The purpose of the interim analysis is to calculate conditional power and make one of the following decisions: (1) declare efficacy and stop the trial early if strong evidence of efficacy in either study group is expected by the test statistic crossing the early termination efficacy boundaries, (2) continue the trial without any design changes if the conditional power is less than 50%, or (3) re-estimate the sample size and continue the trial if the interim result is promising, such as the conditional power being between 50 and 80% [14] All analyses except that of interim will be conducted using two-sided 5% level of significance. Details will be provided in the statistical analysis plan (SAP).

### Study organization and funding

The STACE trial steering committee, chaired by Principal Investigator Bae HJ, is responsible for overseeing all aspects of the trial, ensuring participant safety and managing trial operations. The committee includes key members from the CRCS-K-NIH registry and trial advisors, who work closely with participating centers and the Biostatistics Department at Korea University Graduate School for trial management.

Upon trial completion, the principal investigator and the steering committee will have full access to the dataset for analysis and publication. The trial is funded by Dong-A ST and partially supported by a grant from the Korea National Institute of Health (Project No. 2023-ER-1006-01). The funders have no role in the study’s design, conduct, analysis, interpretation, or reporting of the trial results.

## Discussion

Acute CES patients generally experience more severe strokes and higher mortality rates compared to those with other ischemic stroke etiologies. A previous observational study using the CRCS-K-NIH registry suggested that statin therapy may reduce major vascular events in acute CES patients, even in the absence of guideline-based indications. However, the limitations of nonrandomized studies—such as unmeasured confounding, selection bias, and inaccuracies in medication adherence—necessitate caution in interpreting these results. This trial seeks to address these limitations and definitively assess whether statin therapy improves major clinical outcomes in acute CES patients without indications for its use.

Regardless of the trial’s results, the findings will provide valuable insights into the role of statin therapy in acute CES patients without clear indications. A positive result could support the expansion of statin therapy indications, potentially improving outcomes for stroke patients. On the other hand, a negative result may help clarify the potential overuse of statins when guidelines and clinical performance measures are misaligned.

## Trial status

The latest version of the trial protocol is 1.6, dated 23 December 2024. The trial began recruiting patients on 1 December 2022 and is actively enrolling. As of May 31, 2025, 375 patients (36.2% of the planned sample size) have been enrolled.

## Supplementary Information

Supplementary Material 1.

## Data Availability

At present, data sharing is not available. Following the completion of the trial, data access may be considered for scientifically valid requests, subject to review and approval by the investigators from the participating centers.

## Abbreviations

TIA: Transient ischemic attack
CHD: Coronary heart disease
CES: Cardioembolic stroke
SPARCL: The Stroke Prevention by Aggressive Reduction in Cholesterol Levels
CRCS-K-NIH: Clinical Research Collaboration in Korea-National Institute of Health
STACE: The Statin Therapy for the Prevention of Major Clinical Events in Acute CardioEmbolic Stroke Patients without a Definite Indication for Statins
PROBE: Prospective, randomized, open-label, blinded end-point
RRCT: Registry-based randomized clinical trial
ACC: American College of Cardiology
AHA: American Heart Association
ASCVD: Atherosclerotic cardiovascular disease
LDL-C: Low-density lipoprotein-cholesterol
DSMB: Data and Safety Monitoring Board
ITT: Intention-to-treat
PP: Per-protocol
