# Supplementary material for: Effect of atorvastatin versus no Statin Treatment on major clinical events in Acute CardioEmbolic stroke patients without a definite indication for statin therapy: protocol for the STACE trial
Source: Trials. 2025 Sep 26;26:358. doi: 10.1186/s13063-025-09097-x (PMC12465799; doi:10.1186/s13063-025-09097-x)
Supplement: Supplementary file 1 — Supplementary Material 1. [file 13063_2025_9097_MOESM1_ESM.docx]

**ONLINE SUPPLEMENTS**

Title: Statin therapy on prevention for composite event outcome in acute cardioembolic stroke patients with no definite indication for statin (STACE) trial: Rationale and design

**Supplemental Methods**

**Supplemental Tables.**

Supplemental Table I. List of high-risk sources of cardioembolism

Supplemental Table II. Outcome capture process with questionnaires

Supplemental Table III. Definitions of outcome variables in the CRCS-K registry and STACE trial.

Supplemental Table IV. Randomization scenarios for the STACE trial

Supplemental Table V. The result of 10 times of simulation for scenario #3.

**Supplemental Reference: 1**

**Supplemental Methods**

***Study design***

CRCS-K registry

The CRCS-K registry is a nationwide, multicenter, prospective, web-based registry of consecutive patients with acute ischemic stroke or transient ischemic attack (TIA) admitted to 20 academic hospitals or regional stroke centers in South Korea ([http://www.stroke-crc.or.kr/ecrf](about:blank)). The database contains demographic including age, sex, and prestrike functional status; medical history such as smoking status, previous stroke or TIA, hypertension, diabetes mellitus, dyslipidemia, atrial fibrillation, symptomatic carotid disease, heart failure, coronary heart disease (acute coronary syndrome, angina, and coronary revascularization), and cardiac sources of embolism; laboratory data including total cholesterol, triglycerides, high-density lipoprotein cholesterol, low-density lipoprotein cholesterol, fasting glucose, hemoglobin A1c, systolic and diastolic blood pressure; stroke characteristics such as initial stroke severity measured with the National Institute Health Stroke Scale score, presence of steno-occlusion of relevant arteries; treatments including hyperacute reperfusion therapy (intravenous thrombolysis and endovascular therapy), antithrombotic drugs, and statin therapy. Approximately 7,500 patients have been enrolled annually, and more than 95% of them have completed the 3-months and 1-year outcome captures. The central data manager monitors the cases and their data quality biweekly to minimize any bias or missing data. Pre-specified queries are used, and peripheral registrars revise erroneous entries upon inquiries.

Registry-based randomized controlled trial (RRCT)

The RRCT design offers several advantages in clinical research. By utilizing real-world patient data, it ensures diverse patient representation, thereby enhancing the generalizability of the findings.^1^ Integrating randomization within registry frameworks increases trial efficiency, minimizes recruitment challenges, and reduces administrative costs. Additionally, since registry data are collected as part of routine clinical practice, the effort required to capture outcomes is reduced compared to traditional RCTs. Overall, the registry-based RCT approach provides valuable insights into treatment effectiveness and safety in real-world settings, offering a cost-effective and efficient method for conducting clinical research.

Population

*Four statin benefit groups*

The four statin benefit groups are: 1) individuals with clinical atherosclerotic cardiovascular disease (ASCVD) (including acute coronary syndrome, history of myocardial infarction, stable or unstable angina, coronary or other arterial revascularization, and stroke, TIA, or peripheral arterial disease presumed to be of atherosclerotic origin; 2) individuals with elevation of low-density lipoprotein-cholesterol (LDL-C) ≥190 mg/dL; 3) individuals 40-75 years of age with diabetes, and LDL-C 70-189 mg/dL without clinical ASCVD; or 4) individuals without clinical ASCVD or diabetes, who are 40-75 years of age with LDL-C 70-189 mg/dL, and have an estimated 10-year ASCVD risk of 7.5% or higher.

Medication Adherence

Medication adherence in the statin user group is assessed at the 3-month follow-up using a combination of the 6-item Morisky Medication Adherence Scale (MMAS-6) and patient-reported adherence rates, defined as the proportion of prescribed doses the patient reports having taken during the study period.

Patients in the statin non-user group do not undergo formal adherence scoring; however, they receive repeated education throughout the study period to avoid inadvertent statin use, including during visits to other departments. Investigators explicitly instruct both participants and caregivers to refrain from initiating statins during the 3-month intervention period.

**Supplemental Table I.** List of high-risk sources of cardioembolism

| High-risk sources of cardioembolism |
| --- |
| Atrial fibrillation / Paroxysmal atrial fibrillation  Sustained atrial flutter  Sick sinus syndrome  Left atrial, atrial appendage, or ventricular thrombus  Mechanical prosthetic valve  Dilated cardiomyopathy  Myocardial infarction  Recent (within 1 month)  Chronic, with low left ventricular ejection fraction (<28%) |

**Supplemental Table II.** Outcome capture process with questionnaires.

| - **Chart review**   Last clinic visit: _____/___/___ (YYYY/MM/DD) Capture date: _____/___/___ (YYYY/MM/DD)  Follow-up: □ Our hospital □ Outside hospital/clinic □ No follow-up  #1. Death after discharge  ○ YES  □ Death with obvious cause / during admission 🡪 Finish after chart review  1) Date of death: _____/___/___ (YYYY/MM/DD)  2) Cause of death:  ○ Stroke death: within 4 weeks prior to death  □ Ischemic □ Hemorrhagic □ Unknown  ○ Cardiovascular death (□ MI □ Heart failure □ Other vascular cause*)  (*: arrhythmia, pulmonary thromboembolism, systemic bleeding, vascular disease in major organs or extremities)  ○ Other death  □ Infection □ Extracranial bleeding □ Cancer □ Trauma □ etc: ______ □ Unknown  □ Death on arrival, uncertain cause of death 🡪 Go to #4 after the chart review  ○ NO 🡪 Go to #5 after the chart review  #2. Stroke recurrence after discharge: ○ YES ○ NO  1) If yes, how many times have you heard?: □ 1 □ 2 □3  2) Type of stroke: □ Ischemic □ Hemorrhagic □ TIA □ Unknown  3) Date of stroke recurrence: _____/___/___ (YYYY/MM/DD) (□ within 3 weeks □ after 3 weeks)  4) if stroke was recurred within 3 weeks after discharge, was there any following condition?  i) New neurological symptoms/signs or any neurological worsening after a period of neurological stabilization or improvement lasting for 24 hours or more: □ Yes □ No  ii) Not attributable to perilesional edema, mass effect, brain shift syndrome, or hemorrhagic transformation of the index stroke, or other medical condition: □ Yes □ No  iii) New neurological symptoms/signs or any neurological worsening with newly detected lesions on the CT or the MR: □ Yes □ No  5) Laterality of stroke: if the patient has the image regarding the stroke recurrence  5-1) Check the lesion of the index stroke   \| By territory \| Lt \| Rt \| Both \| \| --- \| --- \| --- \| --- \| \| ICA \| □ \| □ \| □ \| \| MCA \| □ \| □ \| □ \| \| ACA \| □ \| □ \| □ \| \| PCA \| □ \| □ \| □ \| \| BA \| □ \| □ \| □ \| \| VA \| □ \| □ \| □ \| \| SCA \| □ \| □ \| □ \| \| AICA \| □ \| □ \| □ \| \| PICA \| □ \| □ \| □ \| \| Negative \| □ \| \| \|   5-2) if the stroke was recurred and the location can be documented, check the lesion of the stroke recurrence   \| By territory \| Lt \| Rt \| Both \| \| --- \| --- \| --- \| --- \| \| ICA \| □ \| □ \| □ \| \| MCA \| □ \| □ \| □ \| \| ACA \| □ \| □ \| □ \| \| PCA \| □ \| □ \| □ \| \| BA \| □ \| □ \| □ \| \| VA \| □ \| □ \| □ \| \| SCA \| □ \| □ \| □ \| \| AICA \| □ \| □ \| □ \| \| PICA \| □ \| □ \| □ \| \| Negative \| □ \| \| \|   ① Is the lesion corresponding to the symptom? □ Yes □ No  ② If there is no new lesion, does the symptom match the definition of stroke? □ Yes □ No  #3. Cardiac disease: ○ YES ○ NO  1) If yes, number of cardiac disease: □ 1 □ 2 □3  2) Type of cardiac disease: □ Angina □ AMI □ Congestive heart failure □ Unknown  3) Date of cardiac disease: _____/___/___ (YYYY/MM/DD)  4) If the MI occurred, check among the followings:  □ Typical symptoms of myocardial infarction (chest pain, etc) □ Enzyme changes indicative of MI □ ECG changes indicative of MI  **Caution before the telephone interview**  Try to make a close and harmonious relationship before the telephone interview using introduction and greeting. Example is following: Hello, this is OOO, stroke nurse, working at XXX hospital. I am calling you to evaluate how much you are improved 3 months (1 year) after the stroke, and to ask several question. How have you been doing after discharge? Do you have some time? Then, I am going to ask you several questions that are related to your medical conditions and healthcare activities after discharge. If you have any question during the interview, be free to ask any time.   - **Interview (3 months / 1 year)**   First abnormal time: ____/__/__ (YYYY/MM/DD) Discharge date: ____/__/__ (YYYY/MM/DD)  Capture date: _____/___/___ (YYYY/MM/DD) Information provider: □ Patient □ Guardian  Contact loss: □ unable to contact (not responding to call) □ refusal □ death  #4. Death on arrival, uncertain cause of death  #4-1. Have you ever heard that he (or she) had had a stroke “recurrence” from the doctor, or the symptoms indicating stroke appeared and disappeared within 24 hours after discharge? □ Yes □ No  1) If yes, how many times have you heard?: □ 1 □ 2 □3  2) Type of stroke: □ Ischemic □ Hemorrhagic □ TIA □ Unknown  3) Date of stroke recurrence: _____/___/___ (YYYY/MM/DD) (□ within 3 weeks □ after 3 weeks  4) What kinds of symptoms occurred? (check all)  □ Unilateral motor weakness of extremities  □ Unilateral sensory disturbance of extremities  □ Loss of vision  □ Dysarthria  □ Inability to comprehend or formulate language  □ Diplopia  □ Disequilibrium or dizziness  □ Other ( )  5) Where was the stroke recurrence diagnosed? (name of the hospital/clinic: )  #4-2. Have you ever heard that he (or she) had had a cardiac disease from the doctor before his (or her) decease? □ Yes □ No  1) If yes, how many times have you heard?: □ 1 □ 2 □3  2) Type of cardiac disease: □ Angina □ AMI □ Congestive heart failure □ Other vascular cause*  (*: arrhythmia, pulmonary thromboembolism, systemic bleeding, vascular disease in major organs or extremities)  3) Date of cardiac disease: _____/___/___ (YYYY/MM/DD)  4) Where was the cardiac disease diagnosed?  - If any event was occurred more than one time, construct another form for the event.  #5. The patient is alive according to the medical record review  #5-1. Where are you living now? □ Hospital □ Home □ Nursing home □ Death  1) If the patient died, when was it? _____/___ (YYYY/MM)  2) What was the cause of death?  i) Was the stroke the cause of death? □ Yes □ No  - Type of stroke: □ Ischemic □ Hemorrhagic □ Unknown  ii) Was the cardiac disease the cause of death? □ Yes □ No  - Type of cardiac disease: □ AMI □ Congestive heart failure □ Other vascular cause*  (*: arrhythmia, pulmonary thromboembolism, systemic bleeding, vascular disease in major organs or extremities)  iii) Other death (□ Infection □ Extracranial bleeding □ Cancer □ Trauma □ etc:_____________ □ Unknown)  #5-2. mRS score: _________ (0~6, 9 *9=follow-up loss)  #5-3. Do you take medicine these days? □ Yes 🡪 1), 2) □ No 🡪 3) □ Not available  1) Do you ever forget to take your medicine? □ Yes □ No  Are you careless at times about taking your medicine? □ Yes □ No  When you feel better do you sometimes stop taking your medicine? □ Yes □ No  Sometimes if you feel worse when you take your medicine, do you stop taking it? □ Yes □ No  Do you know the long-term benefit of taking your medicine as told to you by your doctor or pharmacist? □ Yes □ No  Sometimes do you forget to refill your prescription medicine on time? □ Yes □ No  2) Please rate your adherence to medicine from 0 to 100. _______  3) What was the reason for not taking medicine? □ Cost □ Adverse effect □ No effect □ Other ______________________  #5-4. Have you ever heard that you had had a stroke “recurrence” from the doctor, or the symptoms indicating stroke appeared and disappeared within 24 hours after discharge? □ Yes □ No  1) If yes, how many times have you heard?: □ 1 □ 2 □3  2) Type of stroke: □ Ischemic □ Hemorrhagic □ TIA □ Unknown  3) Date of stroke recurrence: _____/___/___ (YYYY/MM/DD) (□ within 3 weeks □ after 3 weeks  4) What kinds of symptoms occurred? (check all)  □ Unilateral motor weakness of extremities  □ Unilateral sensory disturbance of extremities  □ Loss of vision  □ Dysarthria  □ Inability to comprehend or formulate language  □ Diplopia  □ Disequilibrium or dizziness  □ Other ( )  5) Where was the stroke recurrence diagnosed? (Name of the hospital/clinic: )  #5-5. Have you ever heard that you had had a cardiac disease from the doctor? □ Yes □ No  1) If yes, how many times have you heard?: □ 1 □ 2 □3  2) Type of cardiac disease: □ Angina □ AMI □ Congestive heart failure □ Other vascular cause*  (*: arrhythmia, pulmonary thromboembolism, systemic bleeding, vascular disease in major organs or extremities)  3) Date of cardiac disease: _____/___/___ (YYYY/MM/DD)  4) Where was the cardiac disease diagnosed? (Name of the hospital/clinic: ) |
| --- | --- | --- | --- | --- | --- | --- | --- | --- | --- | --- | --- | --- | --- | --- | --- | --- | --- | --- | --- | --- | --- | --- | --- | --- | --- | --- | --- | --- | --- | --- | --- | --- | --- | --- | --- | --- | --- | --- | --- | --- | --- | --- | --- | --- | --- | --- | --- | --- | --- | --- | --- | --- | --- | --- | --- | --- | --- | --- | --- | --- | --- | --- | --- | --- | --- | --- | --- | --- | --- | --- | --- | --- | --- | --- | --- | --- | --- | --- | --- | --- | --- | --- | --- | --- | --- | --- | --- | --- |

**Supplemental Table III.** Definitions of outcome variables in the CRCS-K registry and STACE trial.

| Outcome Event | Definition | Operational definition |
| --- | --- | --- |
| Early neurologic deterioration (END) | Any new neurological symptoms/signs or neurological worsening within 3 weeks of index stroke  Causes of END • Recurrent stroke • Stroke progression • Symptomatic hemorrhagic transformation • Others (deep vein thrombosis, pulmonary embolism, myocardial infarction, etc.)  • Unknown | Any of the following; 1) Increase in total NIHSS score ≥ 2  2) Increase in NIHSS subscores 1a, 1b, or 1c (level of consciousness) ≥ 1  3) Increase in NIHSS subscores 5a, 5b, 6a, or 6b (motor) ≥ 1 4) Any new neurological deficit (even unmeasurable by NIHSS scores) |
| Recurrent stroke for END  (within 3 weeks of index stroke) | Development of END associated with new lesions documented by relevant neuroimaging study | • Discrete new lesions documented by DWI or CT  • If discrete, new lesions within the vascular territory of the index stroke lesion may be counted  • Do not count for increased volume of the index stroke lesions  • Do not count for edema, mass effect, herniation, or hemorrhagic transformation of the index stroke lesions |
| Recurrent stroke  (late recurrence ≥3 weeks following index stroke) | Rapidly developing clinical signs of focal (or global) disturbance of cerebral function, with symptoms lasting 24 hours or longer or leading to death, with no apparent cause other than of vascular origin | Data collected through face-to-face or telephone interview with the patient or next of kin  Question: Were you diagnosed with ischemic stroke or hemorrhagic stroke by any doctor after discharge? |
| Stroke progression | END event in neurologically stable patients ≥ 24 hours  • Not attributable to peri-lesional edema  • For cases with ≤24 hours after onset, END events not attributable to recurrent stroke or peri-lesional edema |  |
| Symptomatic hemorrhagic transformation | END events attributable to documented hemorrhagic transformation and associated with NIHSS score increase ≥ 4 points |  |
| Myocardial infarction (MI) |  | 1) For patients ≤ 3 weeks after index stroke;  More than two from below;  _Typical chest pain _Troponin elevation _ECG changes (new ST segment changes, new Q wave, or new LBBB) 2) For patients ≥ 3 weeks after index stroke;  Data collected through face-to-face or telephone interview with the patient or next of kin. Question: Were you diagnosed with MI by any doctors after discharge? |
| Vascular death | Death due to stroke, MI, or sudden death | Data collected through face-to-face or telephone interview with the patient or next of kin.  No known non-atherosclerotic cause and definite MI or stroke within 4 weeks before death.  No known non-atherosclerotic cause and one or both of the following: chest pain within 72 hours of death or a history of chronic ischemic heart disease (in the absence of valvular heart disease or non-ischemic cardiomyopathy).  No known non-atherosclerotic cause and death certificate consistent with CHD as underlying cause. |
| Non-vascular death | Death not attributable to stroke, MI, or sudden death | Data collected through face-to-face or telephone interview with the patient or next of kin |

END, early neurological deterioration; NIHSS, National Institute of Health Stroke Scale; ECG, electrocardiography; MI, myocardial infarction; CHD, coronary heart disease.

^*^Reference: J Stroke 2015;17:38-53.

**Supplemental Table IV.** Randomization scenarios for the STACE trial

| Days of arrival from symptom onset | The proportion of patients randomly assigned | | | |
| --- | --- | --- | --- | --- |
| Scenario #1 (Very impossible but best case scenario) | At the day of arrival | One day after arrival | Two days after arrival | Three days after arrival |
| Day 0 | 90% | 5% | 5% |  |
| Day 1 | 95% | 5% |  |  |
| Day 2 | 95% | 5% |  |  |
| Scenario #2 (Impossible but better case scenario) |  |  |  |  |
| Day 0 | 50% | 30% | 15% | 15% |
| Day 1 | 70% | 20% | 10% |  |
| Day 2 | 70% | 30% |  |  |
| Scenario #3 (Possible and practical scenario) |  |  |  |  |
| Day 0 | 15% | 50% | 25% | 10% |
| Day 1 | 30% | 55% | 15% |  |
| Day 2 | 30% | 70% |  |  |
| Scenario #4 (Bad and hope not to happen scenario) |  |  |  |  |
| Day 0 | 5% | 15% | 30% | 50% |
| Day 1 | 5% | 25% | 70% |  |
| Day 2 | 5% | 95% |  |  |
| Scenario #5 (Very bad and should not be happened scenario) |  |  |  |  |
| Day 0 | - | 5% | 5% | 90% |
| Day 1 | - | 5% | 95% |  |
| Day 2 | - | 100% |  |  |

**Supplemental Table V.** The result of 10 times of simulation for scenario #3.

| Scenario #3 | Number of patients used for simulations | | | | Cumulative incidence of the primary outcome within 90 days of stroke onset | | |
| --- | --- | --- | --- | --- | --- | --- | --- |
|  | Total | Day 0***** | Day 1**†** | Day 2**‡** | Statin user group | Statin non-user group | Difference |
| Total | 4,859/5,261 (92.4%) | 4,165 | 706 | 222 | **6.1% (0.32)**§ | **23.3% (0.48)**§ | **17.2% (0.32)**§ |
| Mild  (NIHSS 0~4) | 1,522/1,608 (94.7%) | 1,156 | 320 | 132 | **5%** | **7%** | **2%** |
| Moderate  (NIHSS 5~10) | 999/1,089 (91.7%) | 793 | 160 | 46 | **7%** | **12%** | **5%** |
| Severe  (NIHSS > 10) | 2,337/2,564 (91.1%) | 2,079 | 216 | 42 | **8%** | **33%** | **25%** |

*****Day 0: Arrival at the day of symptom onset

**†**Day 1: Arrival 1 day after symptom onset

**‡**Day 2: Arrival 2 days after symptom onset

§Mean (standard deviation) obtained from 10 times of simulation.

**Supplemental Reference**

1. Ford I, Norrie J. Pragmatic Trials. *N Engl J Med*. 2016;375:454-463. doi: 10.1056/NEJMra1510059
